# Nepenthesin Protease Activity Indicates Digestive Fluid Dynamics in Carnivorous *Nepenthes* Plants

**DOI:** 10.1371/journal.pone.0118853

**Published:** 2015-03-09

**Authors:** Franziska Buch, Wendy E. Kaman, Floris J. Bikker, Ayufu Yilamujiang, Axel Mithöfer

**Affiliations:** 1 Department of Bioorganic Chemistry, Max Planck Institute for Chemical Ecology, Hans Knöll Straße 8, D-07745, Jena, Germany; 2 Department of Medical Microbiology and Infectious Diseases, Erasmus MC, `s-Gravendijkwal 230, 3015 CE, Rotterdam, The Netherlands; 3 Department of Oral Biochemistry, Academic Centre for Dentistry Amsterdam, University of Amsterdam and VU University Amsterdam, Gustav Mahlerlaan 3004, 1081 LA, Amsterdam, The Netherlands; Pennsylvania State University, UNITED STATES

## Abstract

Carnivorous plants use different morphological features to attract, trap and digest prey, mainly insects. Plants from the genus *Nepenthes* possess specialized leaves called pitchers that function as pitfall-traps. These pitchers are filled with a digestive fluid that is generated by the plants themselves. In order to digest caught prey in their pitchers, *Nepenthes* plants produce various hydrolytic enzymes including aspartic proteases, nepenthesins (Nep). Knowledge about the generation and induction of these proteases is limited. Here, by employing a FRET (fluorescent resonance energy transfer)-based technique that uses a synthetic fluorescent substrate an easy and rapid detection of protease activities in the digestive fluids of various *Nepenthes* species was feasible. Biochemical studies and the heterologously expressed Nep II from *Nepenthes mirabilis* proved that the proteolytic activity relied on aspartic proteases, however an acid-mediated auto-activation mechanism was necessary. Employing the FRET-based approach, the induction and dynamics of nepenthesin in the digestive pitcher fluid of various *Nepenthes* plants could be studied directly with insect (*Drosophila melanogaster*) prey or plant material. Moreover, we observed that proteolytic activity was induced by the phytohormone jasmonic acid but not by salicylic acid suggesting that jasmonate-dependent signaling pathways are involved in plant carnivory.

## Introduction

Charles Darwin’s work “Insectivorous Plants” published in 1875 [1] still contains much of what we know about those specialized plants. However, Darwin never worked with the genus *Nepenthes*, which is distributed primarily in Southeast Asia. *Nepenthes* pitcher plants have so-called pitfall-traps that are divided into *i)* an upper part representing the attraction zone, *ii)* a part in the middle representing the slippery zone, and *iii)* a lower part, the digestion zone. Pitchers are filled with a digestive fluid, or enzyme cocktail, to digest caught prey [2,3]. Even closed pitchers have such a fluid, which is both plant-derived and sterile [4]. Since Darwin, scientists have known that hydrolytic activity—in particular, proteolytic activity—is present in insectivorous plants. In addition to proteases, the digestive fluid of *Nepenthes* spp. is known to contain various esterases, phosphatases, ribonucleases and different chitinases (e.g. [2,3,5,6,7,8]). Proteases in digestive fluid from several species of *Nepenthes* have also been described early [9], purified and characterized (e.g. [10,11,12]). However, only An et al.[13] cloned nepenthesins from the pitcher tissue of *N*. *alata*. Two years later nepenthesin I and II from *N*. *distillatoria* were purified and characterized [14]. After the nepenthesin cDNAs were cloned from *N*. *gracilis* pitchers [14], these proteases were identified as members of a new subfamily of aspartic proteases [14,15,16]. In addition, Stephenson and Hogan [17] reported a cysteine protease in *N*. *ventricosa*.

Otherwise, little is known about the regulation and induction of hydrolytic enzymes involved in the digestive process in carnivorous plants. In recent years, gene induction analyses were carried out on the tissue of the pitcher to search for hydrolytic enzymes [5,6,18]. This also holds true for the very prominent aspartic proteases in *Nepenthes* [13], although the proteolytic activity in the pitcher fluid represents an ideal target to follow and study dynamic processes during carnivory in *Nepenthes* pitfalls. But up to now, the low amounts of enzymes in the pitchers have made it impossible to analyze changes in the digestive fluid depending on developmental stages of the pitcher or in response to prey capture.

Here, we report on the introduction of a new technique, the highly sensitive FRET (fluorescent resonance energy transfer), for the direct, easy and rapid detection and characterization of protease activity in the digestive fluids of *Nepenthes*. Using a synthetic fluorogenic substrate, i.e. FRET peptide, we investigated the dynamics of protease activity in response to various stimuli. In addition, we cloned and expressed the proteases involved in the enzymatic reaction, nepenthesin I and II.

## Material and Methods

### Organisms and culture conditions

*Nepenthes* plants *(N*. *mirabilis*, *N*. *alata)* were grown in the greenhouse of the Max Planck Institute for Chemical Ecology in Jena under controlled conditions. The plants were cultivated in a growth chamber with a photoperiod of 15 h light/9 h dark, day/night temperature of 18–20°C/16–18°C and humidity about 55%. Every day, plants were sprayed and every second day they were watered with rain water.

Both tissue from the lower part of the pitchers and pitcher fluid from *N*. *mirabilis* and *N*. *alata* were used for this study. As well, the pitcher fluid of other *Nepenthe*s species (*N*. *reinwardtiana*, *N*. *distillatoria*, *N*. *wittei*, *N*. *hookariana*, *N*. *boschiana*, *N*. *maxima*, *N*. *eymae* and the hybrid *N*. *alata x N*. *ventricosa*), which were grown in the greenhouses of the Botanical Gardens in Jena and Munich, were used for fluorescence intensity measurements. The pitcher fluid samples were collected from closed pitchers using a sterile syringe, from open pitchers by pouring fluid directly into 15 ml sterile Falcon tubes.

*Spodoptera frugiperda* Sf9 cells, derived from the pupal ovarian tissue of the insect and originating from the IPLBSF-21 cell line (Invitrogen, Darmstadt, Germany), were used for the transfection and expression of the *Nepenthes* aspartic proteinases, nepenthesin (Nep) I and II. They were cultured at 27°C in Sf-900 II serum-free medium (sf- medium) (Gibco) in presence of 50 μg/ml gentamycin.

### Measuring protease activity with fluorescent substrate and FRET

Using a small and highly specific FRET peptide substrate (FITC(Ahx)-Val-Val-LysDbc), encoded as PFU-093 by Kaman et al. [19,20], we measured the proteolytic activity of the pitcher fluid. PFU-093, one of many substrates developed to study the presence of bacteria *in situ* (saliva, sputa, serum), was designed with fluorescein isothiocyanate (FITC) operating as a fluorophore and LysDbc acting as its quencher. When both molecules are physically close, the connection made by the two-amino acid bridge quenches the fluorescence and no activity can be detected (Fig. Aa in S1 File). However, when proteolytic activity separates the fluorophore and quencher, fluorescence intensity can be measured using a microplate reader (Tecan infinite M200, Männedorf, Switzerland) (Fig. Ab in S1 File). We mixed 50 μl of *Nepenthes* pitcher fluid, 49 μl pure water (Gibco) for dilution and 1 μl of 80 μM PFU-093 in black 96-well microtiter plates (Greiner Bio-one GmbH, Frickenhausen, Germany), and measurements were taken for up to 11 h. The fluorescence activity was measured at 42°C and an excitation/ emission wavelength of 485 nm/530 nm.

### Biochemical studies

The impact of pH on the FRET method was tested by incubating 500 μl *N*. *alata* pitcher fluid mixed with 245 μl pure water and 5 μl of 80 μM PFU-093. After 10 h at 42°C, 50 μl each of this mixture was transferred into 10 different wells and mixed with another 50 μl of various 30 mM buffer solutions (pH 2, pH 3, pH 4, up to pH 10) with H_2_O as a control. Then fluorescence was measured and the following categories were used: for an acidic range, citrate buffer from pH 2 to 4.9; for an acid-base balance, phosphate buffer from pH 4.9 to pH 8.5; and for a basic pH range, glycine buffer from pH 8.5–10.

To verify the stability of the reaction, digestive fluid was pre-incubated with 30 mM citrate buffer pH 4.0 for 10 h at 42°C; as a control, pre-incubation was done with water. For fluorescence measuring, 50 μl of this mixture was either mixed with 30 mM phosphate buffer pH 8 or with 30 mM citrate buffer pH 4.

Inhibitor tests were performed by mixing 50 μl of pitcher fluid, 1 μl of 80 μM PFU-093 and various inhibitors at different concentrations: including 100 μM phenanthroline, 100 μM AEBSF, 20 μM E-64 and 100 μM pepstatin A. Samples were mixed with pure water to 100 μl final volume and measured as described earlier, including the inhibitors solvents (DMSO, MeOH, H2O) and pure pitcher fluid for control.

### Determination of substrate cleavage site

To determine the cleavage site of PFU-093 substrate cleaved by *Nepenthes* aspartic proteases, Nep I/II, HPLC-MS was performed. For this measurement, 495 μl pitcher fluid of both *N*. *mirabilis* and *N*. *alata*, as well as 5 μl of 80 μM PFU-093 substrate, was added to 1.5 ml safe-lock tubes (Eppendorf AG, Hamburg, Germany) and incubated for 11 h at 42°C. This mixture was concentrated by drying and subsequently resolved in 60 μl H_2_O. Of the concentrated probe, 20 μl was injected into a Dionex UltiMate 3000 HPLC system, equipped with a Kinetex C18 column and connected to a Thermo Fisher LTQ for MS. ESI-MS in positive ion mode was used for searching three different fragment masses; *m/z* 397.47, *m/z* 496.28, *m/z* 619.8 (Fig. Ac in S1 File).

### RNA preparation and cDNA synthesis

Tissue samples were collected by cutting a pitcher for homogenization, using only the lower third, i.e. the part that possesses the multicellular glands [21]. Total RNA from the lower part of one *N*. *mirabilis* pitcher was isolated using the InviTrap Spin Plant RNA Mini Kit (Stratec Molecular, Berlin, Germany) following the manufacturer’s protocol. For RNA cleanup and concentration RNeasy MinElute Cleanup Kit (Qiagen, Hilden, Germany) was used. First-strand cDNA was synthesized using SuperScript III First-Strand Synthesis SuperMix (Invitrogen) as well as up to 5 μg total RNA according to the specified protocol.

### Rapid amplification of cDNA ends (RACE), cloning and sequencing

Previously sequenced *N*. *gracilis* and *N*. *alata* nepenthesin mRNA transcripts (GenBank accessions: *Ng*NepI, AB114914; *Ng*NepII, AB114915; *Na*NepI AB266803) were aligned using the CLUSTAL-W multiple sequence alignment program (available from: http://www.genome.jp/tools/clustalw/) and assessed for percent sequence identity. Specific primers for both nepenthesin I and II were designed in regions where 100% sequence identity was found (Table A in S1 File, primer sequences 1–4). These primers were used to amplify fragments of cDNA sequences using *N*. *mirabilis* cDNA as a template for the PCR reactions. Subsequently, the amplified products were cloned into a pCR 2.1-TOPO vector following the described TOPO TA cloning protocol (Invitrogen, Darmstadt, Germany) and sent for sequencing (Eurofins MWG Operon, Ebersberg, Germany). The resulting partial sequences were used to design *N*. *mirabilis* Nep I and II RACE primers via version 4.0.0. of Primer3Web software [22] (Table A in S1 File, RACE primer sequences 5–8). For generating 5’- and 3’- RACE-Ready cDNA, the manual of the SMARTer RACE cDNA Amplification Kit (Clontech, Mountain View, CA, Canada/ USA) was followed. The generation of 5’ and 3’ cDNA fragments of Nep I and II was described as 5’- RACE and 3’- RACE PCR reactions in the manufacturer’s manual. To clean and concentrate DNA, DNA Clean & Concentrator-5 (Zymo Research, Irvine, CA, USA) was used. The resulting amplified products were cloned into a pCR 2.1-TOPO vector following the described procedure (see above). The resulting plasmids were sequenced by Eurofins MWG Operon. The complete Nep I and II cDNA sequences were identified by using the DNASTAR Lasergene Software SeqMan Pro (Madison, WI, USA). Subsequently, known Nep I and II protein sequences from *N*. *gracilis (Ng)* and *N*. *alata (Na)* were compared with the sequences of *N*. *mirabilis* (*Nm*). Protein sequences from NCBI Genbank were aligned by using “Jalview- *Open Source* Bioinformatics”- Software [23] and “MegAlign (DNASTAR)”- software version 10.1.2.

### Expression in insect cells and Western blot

For functional identification, cDNA was amplified with primer sequences 9–10 for Nep I and primer sequences 11–12 for Nep. II (Table A in S1 File) to obtain an open reading frame (ORF) that lacks the predicted signal peptide (SP). The cDNA from Nep I and II was subcloned in pMIB/V5-His vector A (Invitrogen, Darmstadt, Germany) for transfection into Sf9 cells using lipid-mediated transfection. Cells were transfected in 60-mm diameter Petri dishes with 4 μg of plasmid DNA using Insect Gene Juice (Novagen, Nottingham, UK) as a transfection reagent. After 48 h, cells were split 1:5 in a 60-mm diameter Petri dish and selected with 80 μg/ ml blasticidin until they reached confluency. Expression was analyzed by Western blot using the anti-V5 horseradish peroxidase antibody (Invitrogen).

### Auto-activation of *Nepenthes mirabilis* nepenthesin II

Recombinant nepenthesin II (*Nm*NepII/Sf9) from *N*. *mirabilis*, expressed in Sf9 culture medium, was dialyzed against pure H_2_O (Slide-A-Lyzer Dialysis Cassettes; 10.000 MWCO, Thermo Fisher Scientific, Bonn, Germany) for 24 h; the same was done with Sf9 culture medium (Sf9cm) only for a control. Next, 500 μl of the dialyzed *Nm*NepII/Sf9 and 500 μl of Sf9cm were incubated at room temperature; 60 μl 1 M glycine- HCl- buffer (pH 4) was added to promote auto-cleavage of the protease and to eliminate the pro-peptide (in Nep II protein sequence at position 73) by acidification. After 24 h of incubation at pH 4, 100 μl of 100 mM Tris- HCl buffer pH 8.5 was added to both mixtures to achieve a more basic pH range to measure fluorescence. Furthermore, 500 μl of *Nm*NepII/SF9 and SF9cm was measured without pre-incubation in pH 4 buffer. Fluorescence intensity measurements of each mixture were taken every 5 min for 6 h in a microplate reader in a black 96-well plate with 5 technical replicates.

### Protease activities in *Nepenthe*s pitcher fluids induced by different treatments

To induce protease activity, the pitcher fluids of two *Nepenthes* species, *N*. *alata* and *N*. *mirabilis*, were supplemented with *i)* fruit flies, *Drosophila melanogaster*, as insects represent the natural food, and *ii)* a piece of *Nepenthes* leaf as plant-derived food material. In addition, the phytohormones *iii)* jasmonic acid (JA), and *iiii)* salicylic acid (SA) were also tested for their ability to induce protease activity. In both cases the phytohormones were injected directly into the pitcher fluid.

**Fig 1:**
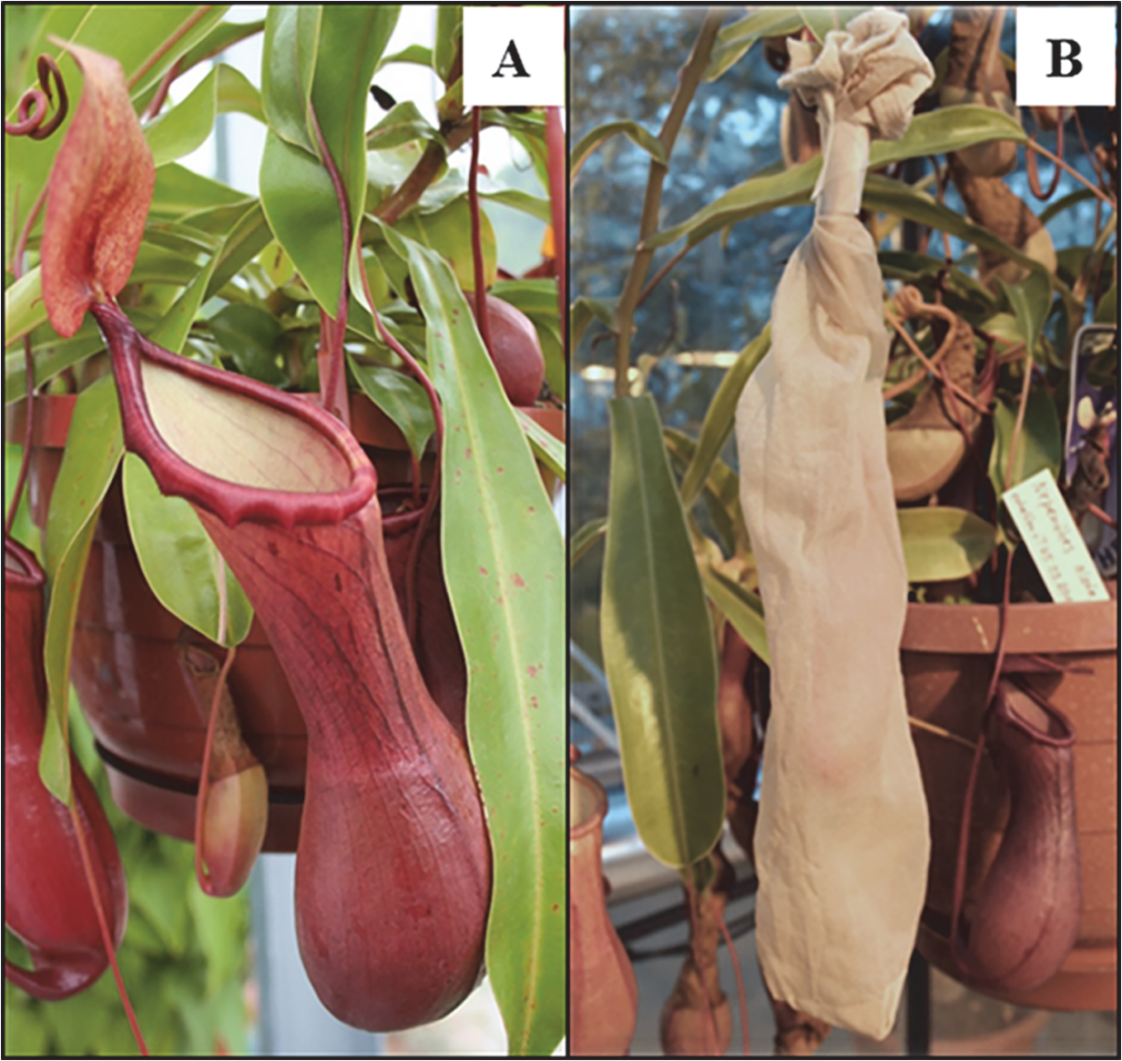
*Nepenthes alata* pitchers. **A** Without and **B** covered with gauze. Pitchers/ pitcher fluid of *Nepenthes* species can be kept under semi-sterile conditions by using gauze.

Fluorescence activity was measured first for control values before feeding/ treating *Nepenthes* plants (time point 0 h) and subsequently at different time points (from 24 h to 240 h) samples were taken and analyzed for proteolytic activity using the PFU-093 substrate. Ten *D*. *melanogaster* flies, two pieces of 1.5 x 1.5 cm damaged *Nepenthes* leaf, and approximately 200 μM each of JA and of SA were added to the pitcher fluid. Usually, three replicates were done; duplicates for feeding with *Nepenthes* leaf material, and four repeats in the case of SA. Experiments were performed under semi-sterile conditions by covering the pitchers with gauze before plants opened their lids (Fig. 1).

As an additional experiment, the pH regulation in the digestive fluid was observed *in vivo*: 5 pitchers were each challenged with 40 *D*. *melanogaster* flies. The pH of digestive fluids was measured before (0 h) and at different time points (between 2 and 192 h) after treatment by immersing test strips directly into the pitcher fluid. Measurements included two control pitchers without fruit flies. In addition, 200 μl samples taken at various time points (0, 96, 144, 192 h) were tested for protease activity by incubating 50 μl pitcher fluid mixed with 39 μl H_2_O and 1 μl of 80 μM PFU-093 substrate per reaction for 5 h at room temperature. Afterwards, probes were mixed with 10 μl of 100 mM Tris- HCl buffer, pH 8.5, to a final volume of 100 μl and pH 8 in each sample, and subsequently, samples were measured in a microplate reader. The removed volume was replaced with 25 mM KCl.

## Results and Discussion

### Proteolytic activity in *Nepenthes* pitchers

**Fig 2:**
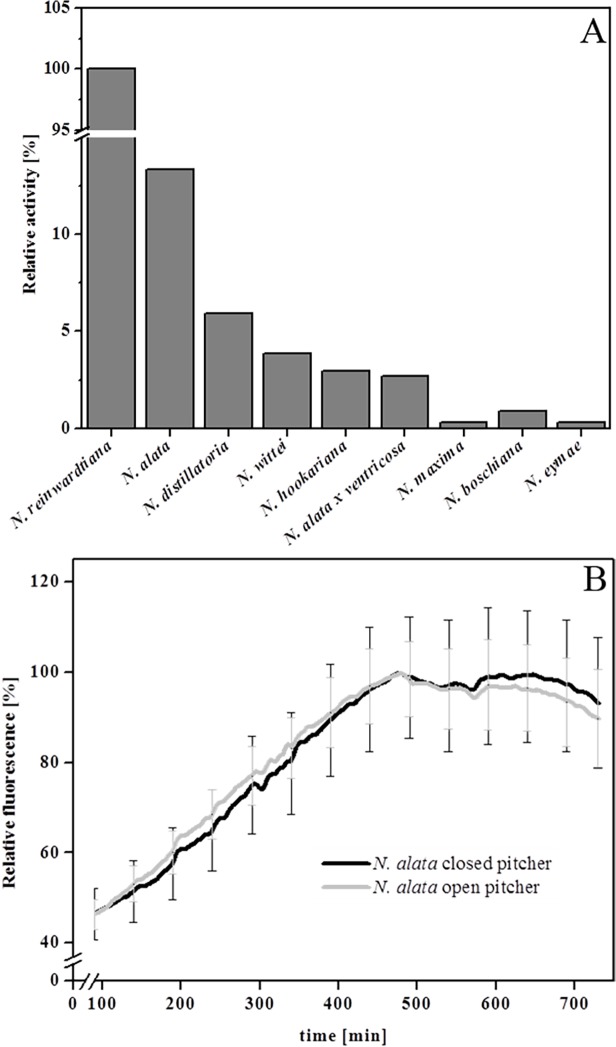
Proteolytic activity in *Nepenthes*. **A** Samples of the pitcher fluid of ten different *Nepenthes* species were investigated for their PFU-093 cleaving activity. **B** Kinetics of proteolytic PFU-093 cleaving activity in pitcher fluid from open (grey line) and closed (black line) pitchers of *Nepenthes alata*. Relative fluorescence was measured over 12 h at 42°C.

Employing the FRET technique with PFU-093 as substrate, proteolytic activity was detected in the pitcher fluid of all ten *Nepenthes* species tested although at different intensities (Fig. 2A). Because fluids from closed pitchers were found to be sterile and contain only plant-derived components [4,24,25], the digestive fluids from closed and open pitchers of *N*. *alata* were also compared. In Fig. 2B, proteolytic activity was detectable in both samples, indicating that the activity in the closed pitcher originated exclusively from the plant itself. Based on that, sterile digestive fluids from closed or newly opened pitchers (kept semi-sterile with gauze, Fig. 1B) were used for further measurements.

The pH of the substrate cleavage reaction was close to 8. This contradicted published results for pitcher fluids containing proteases, describe acidic pH ranges [13,25]. Therefore, the proteolytic reaction was performed at neutral pH (water) for 10 h, and afterwards the pH was adjusted with buffers of high molarity before the final fluorescence was measured. In the control, only water was added. The result (Fig. Ba in S1 File) suggests that the PFU-093 intrinsic fluorescence was quenched under acidic conditions and depended on decreasing pH levels, whereas at neutral and basic pH levels it was detectable. This forced us to keep or adjust the pH value between 7 and 8 in all fluorescence measurements.

In order to analyze whether the proteolytic reaction takes place not only at neutral pH but also under acidic conditions, a subsequent incubation experiment at pH 4 was undertaken. Results revealed that it was possible to restore measurable fluorescence simply by adding a strong buffer and adjusting the pH of the sample to 8 directly after incubation (Fig. Bb in S1 File). This result also demonstrates that the proteases, which cleave the artificial substrate, are stable and active at acidic as well as slightly basic pH ranges, suggesting that these enzymes act like the aspartic proteinases nepenthesin I and II, purified and characterized from several *Nepenthes* species [9,10,11,12,13,14,15,16,17,26].

**Fig 3:**
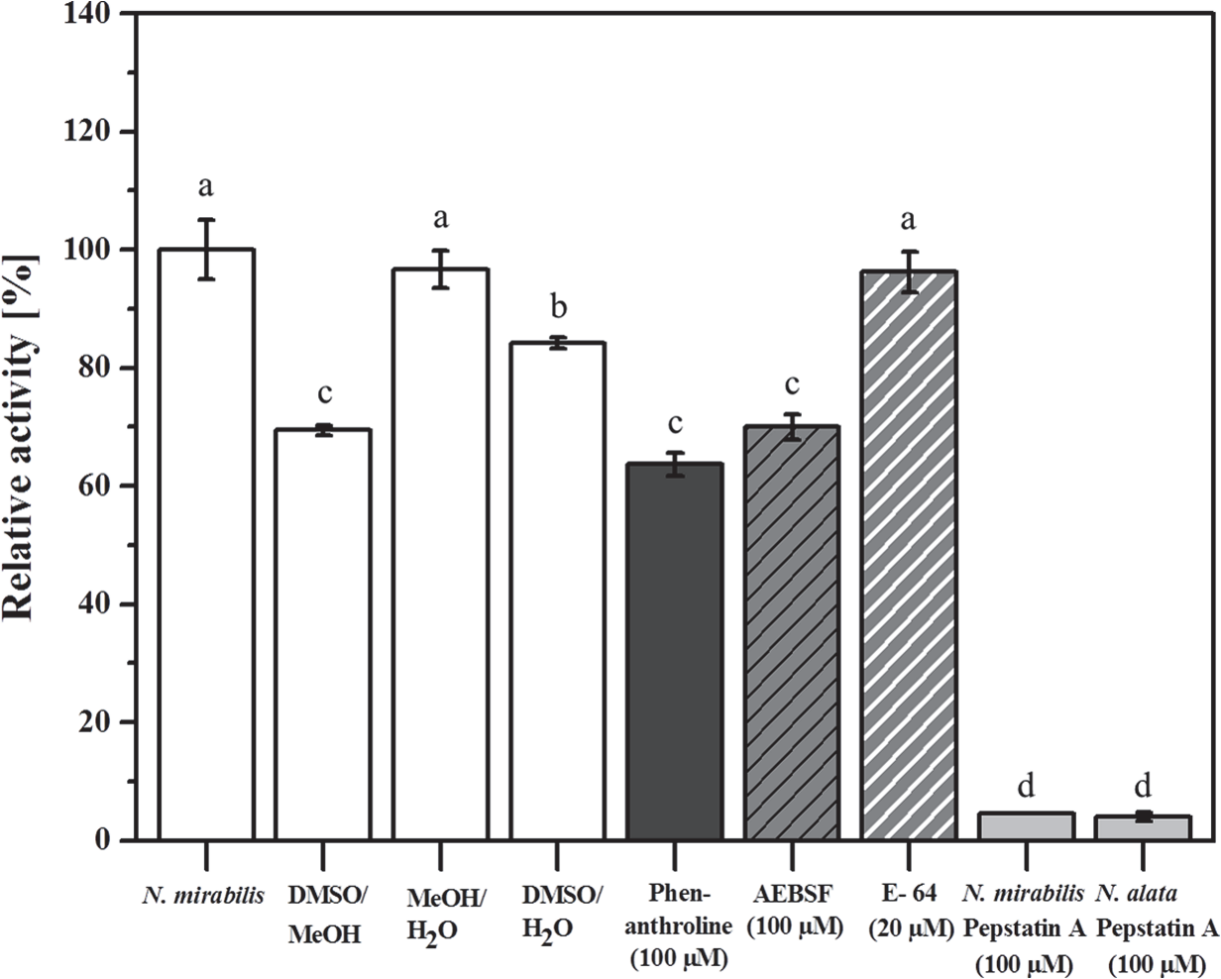
Inhibitor experiments. Different protease inhibitors—phenanthroline, AEBSF, E-64 and pepstatin A—were tested for their ability to inhibit protease activity responsible for PFU-093 cleavage. Individual working concentrations are indicated in brackets. Pitcher fluid from *N*. *mirabilis* without any inhibitor was used as a control (from left, first bar). Additional controls were carried out with the particular solvents of the inhibitors (DMSO, MeOH, H_2_O). Statistics was done using one-way ANOVA, All Pairwise Multiple Comparison Procedures (Student-Newman-Keuls Method), P<0.05; different letters indicate significant differences.

Since the most prominent proteases in *Nepenthes* pitcher fluid are nepenthesins, it was conceivable that the activity we measured was due to those aspartic proteases. To challenge this idea, various inhibitors with specificities against different types of proteases were used in combination with the proteolysis assay. Inhibitors for metalloproteases (phenanthroline), serine-proteases (AEBSF), and cysteine-proteases (E-64) showed no or minor effect (Fig. 3). In contrast, the proteolytic activity in the pitcher fluid is strongly inhibited (4.0 to 4.5% remaining activity) by the aspartic protease inhibitor pepstatin A. This result is in agreement with results obtained for pepstatin-inhibition of nepenthesin activity in *N*. *alata* [13] and *N*. *distillatoria* [14].

**Fig 4:**
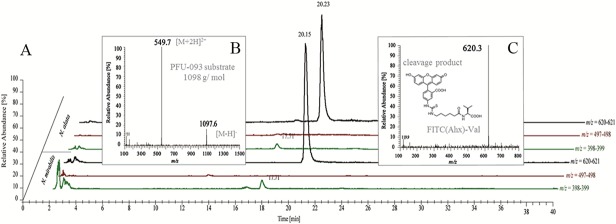
Determination of protease cleavage site. A sample of PFU-093 after digestion with pitcher fluid proteases (*N*. *mirabilis* and *N*. *alata*) was analyzed by HPLC-MS. **A** Single ion chromatograms for mass ranges *m/z* = 620–621 amu (black line), *m/z* = 497–498 amu (red line) and *m/ z* = 398–399 amu (green line). **B** Mass spectrum of PFU-093 (FITC(Ahx)-Val-Val-LysDbc). **C** Mass spectrum of the FITC(Ahx)-Val fragment after cleavage of PFU-093, FITC(Ahx)-Val-Val-LysDbc (MS was done with ESI in the positive ion mode).

A detailed analysis of the cleavage reaction determined the cleavage site in the substrate (Figs. Ab and Ac in S1 File) shows the putative cleavage sites and the resulting products). An HPLC-MS analysis of the PFU-093 cleavage products after treatment with *Nepenthes* proteases proved that, as expected, the substrate was cleaved between the two valines (Fig. 4). Although the molecular weight of the substrate was 1098 g/mol (Fig. 4B), a fragment ion with *m/z* 620, corresponding to the FITC(Ahx)-Val fragment (Fig. 4C), was detected after flies were digested in pitcher fluid from both *N*. *mirabilis* and *N*. *alata* (Fig. 4A). Searching for a fragment *m/z* = 397–398, we found a smaller peak in the chromatogram (Fig. 4B, green line) at R_*t*_ 17.1 min. This peak likely represents the “LysDbc” cleavage fragment and indicates that the second valine was also cleaved off. No fragment representing “Val-LysDbc” (*m/ z* = 497–498) was detected.

### Cloning and heterologous expression of nepenthesin

To confirm that nepenthesin is the active protease involved in PFU-093 degradation, the cDNAs of nepenthesin I and II were cloned. First RNA was isolated from *N*. *mirabilis* pitchers. After synthesizing cDNA, RACE PCR was further used to isolate the missing 5’ and 3’ regions. The resulting complete cDNA sequences of both Nep I and II, which include 5’- and 3’- untranslated regions, had 1570 and 1610 base pairs, respectively. The ORFs for Nep I and II both had 1314 base pairs, encoding 437 amino acid sequences (GenBank accessions AFV26024 (Nep I), AFV26025 (Nep II). Both Nep I and II possess a signal peptide for secretion which is predicted to have 24 amino acids (prediction was made using SignalP 4.1 Server [27]. The predicted molecular weights of Nep I and II without signal peptides were calculated with 43.7 and 43.5 kDa, respectively. In addition, Nep I possesses seven predicted N-glycosylation sites used NetNGlyc 1.0 Server [28], whereas Nep II possesses only two. These observations support the fact that the glycosylation of nepenthesin proteins has been previously observed in *Nepenthes distillatoria* [14].

Percent identity between *N*. *mirabilis* (*Nm*NepI/II), *N*. *alata* (*Na*NepI/II) and *N*. *gracilis* (*Ng*NepI/II) amino acid sequences, was determined by alignment by using the CLUSTAL-W software program (see above) (Fig. C in S1 File). We found that the Nep I and Nep II sequences had rather high percent identity: 94.1% (*Nm*NepI:*Ng*NepI), 99.1% (*Nm*NepI:*Na*NepI) and 96.1% (*Nm*NepII:*Ng*NepII). However, the maximum percent identity is about 66% when sequences of *Nm*NepI are compared to those of *Nm*NepII. In Fig. C in S1 File the predicted signal peptide (SP) cleavage sites,predicted by SignalP- software [27] are shown to be located between amino acid positions 24 and 25: THS/TS. Thus, the active proteins start with the amino acids “NGPS” (Nep I) and “QSSS” (Nep II), respectively. Between the SPs and the active protein, propeptide sequences [14] were detected (Fig. C in S1 File). The *N*. *mirabilis* proteases were found to be typical nepenthesin-aspartic proteases: on one hand they lack the PSI (plant-specific insertion), which is typical for vacuolar APs [29], and on the other hand, they possess a special insertion assigned to residues 148–169 and known as ‘the nepenthesin- type AP (NAP)- specific insertion’ [14]. This insertion contains four additional cysteine residues (arranged pairwise to form disulphide bonds), shown in Fig. C in S1 File as yellow, dark green, light green and orange; these residues contribute to the primary structure [14,15] and precede the tyrosine residue that is shown as a small green box above the sequence. The other two cysteine residues are red and light red. The two catalytic aspartic acid residues are shown as small black boxes.

For heterologous expression in insect Sf9 cells, primers (see above) were used to amplify the ORFs of Nep I and II excluding their native signal peptide and stop codon. These fragments were cloned in frames with the sequence corresponding to the signal peptide from the bee mellitin at the 5’-end and with a V5-(His)_6_ tag at the 3’-end. After 72 h, culture medium was harvested and tested by Western blotting, using an anti-V5 horseradish peroxidase antibody that showed the expression of *Nm*NepII/Sf9 (Fig. D in S1 File) but not of *Nm*NepI/Sf9. However, *Nm*NepII/Sf9 did not show proteolytic activity.

**Fig 5:**
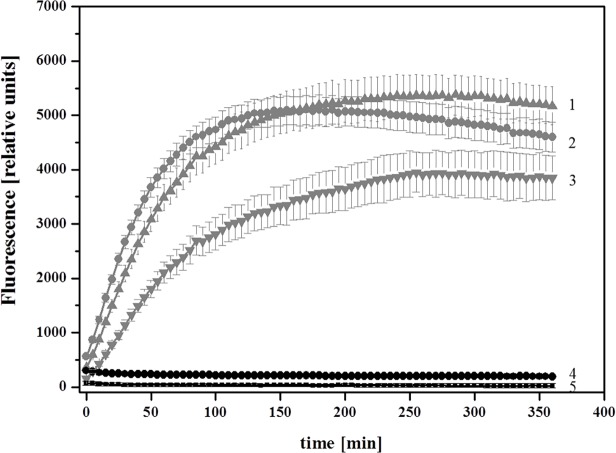
Proteolytic activity of recombinant *N*. *mirabilis* Nepenthesin II (*Nm*NepII/Sf9). PFU-093 fluorescence was measured over 6 h every 5 min after pre-incubation at pH 4 for 24 h for auto-activation. Different concentrations of *Nm*NepII/Sf9 were tested with constant concentrations of PFU-093 substrate. Line 1: 99 μl of *Nm*NepII/Sf9; line 2: 49 μl *Nm*NepII/Sf9; line 3: 24.5 μl *Nm*NepII/Sf9. All were mixed with pure H_2_0 and 1 μl of 80 μM fluorescent substrate to a total volume of 100 μl per well. Controls: line 4: 99 μl *Nm*NepII/Sf9 and H_2_O without the addition of fluorescent substrate; line 5: 99 μl *Nm*NepII/Sf9 and 1 μl of 80 μM PFU-093 without pre-incubation/ auto-activation in pH 4 glycine-buffer.

The proteolytic activity of *Nm*NepII/Sf9 was activated by an acid-mediated cleavage of the expressed propeptide as described for nepenthesin-1 from *N*. *gracilis* expressed in *E*. *coli* [30]. After being activated, *Nm*NepII/Sf9 was incubated for 24h at pH 4; then its protease activity could be measured (Fig. 5) at different protein concentrations (lines 1–3). The control without the presence of PFU-093 substrate (line 4) and the *Nm*NepII/Sf9 control lacking auto-activation (line 5) show no detectable activity. The activity of *Nm*NepII/Sf9 confirms our initial assumption that the aspartic proteases in the pitcher fluid, i.e. nepenthesins, are responsible for cleaving the fluorescent substrate.

**Fig 6:**
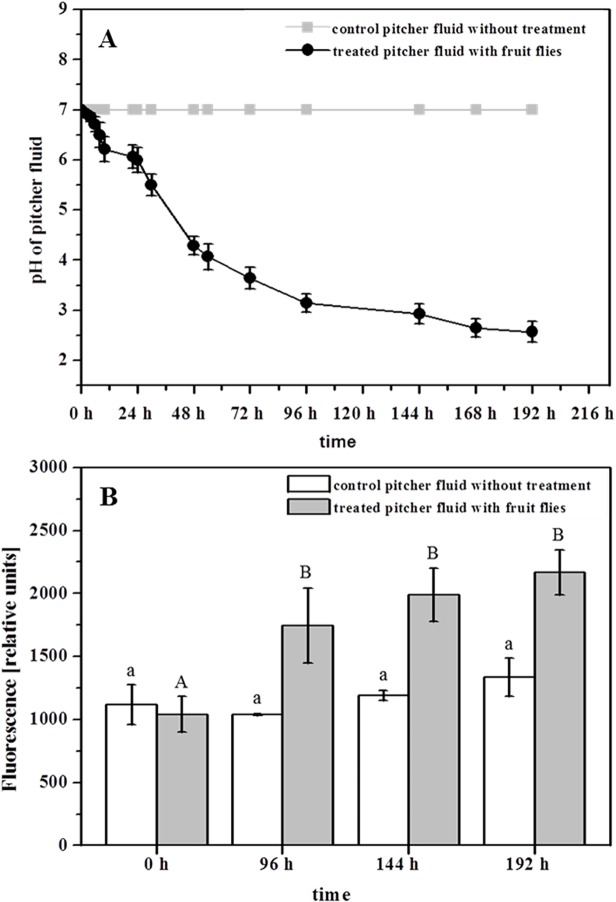
Influence of *Drosophila melanogaster* on pitcher fluid- pH and nepenthesin levels. Pitchers were supplemented with 40 fruit flies each. **A** The pitcher fluid pH was continuously determined at different time points until 192 h after treatment (black line); control without treatment (gray line). **B** Protease activity was measured before (0 h) and after the addition of fruit flies (96, 144, 192 h; gray bars) by using PFU-093 substrate for each sample. Measurement included 2 control pitchers (white bars). For **B** One-way ANOVA, P<0.05, Post Doc Test_*SNK*, was performed.

To see a direct correlation between proteolytic activity and the pH of the pitcher fluid, an additional experiment was done in which pitchers were supplemented with fruit flies and pH was determined every time samples were taken (Fig. 6A). In Fig. 6A, the pH of pitcher fluid decreased after flies were added to the fluid, and a value of 4 was reached in only 48 h, a value of around 3 after 96 h. pH continued decreasing until 192 h. Just as nepenthesins become auto-activated when the surrounding medium is acidified (Fig. 6) [18], so protease activity increases when pitcher fluid is acidified by the addition of *D*. *melanogaster*. In Fig. 6B, a significant increase of protease activity is shown 96 h after the addition of fruit flies, confirming the role of nepenthesins as active proteases.

### Induction of nepenthesins

FRET is a tool to directly analyze proteolytic, namely nepenthesin, levels in the digestive fluids of *Nepenthes*. Compared with others (e.g. [13]), this method is faster, more convenient, and specific for aspartic proteases. Moreover, kinetics can be easily measured with the same sample. The direct analysis of nepenthesins as one of the most prominent enzymes in the digestive fluid of *Nepenthes* pitchers can be seen as the leading-activity for changes in the pitcher fluid activities that occur, for example, after the plants capture prey. As recently shown for the carnivorous plant *Dionaea muscipula* [31,32], besides nepenthesins the presence of more proteases can be expected. However, in *Nepenthes*, only in *N*. *ventricosa* a cysteine proteinase activity was described in the pitcher fluid and a cDNA encoding a putative cysteine protease was cloned from pitcher tissue [17].

**Fig 7:**
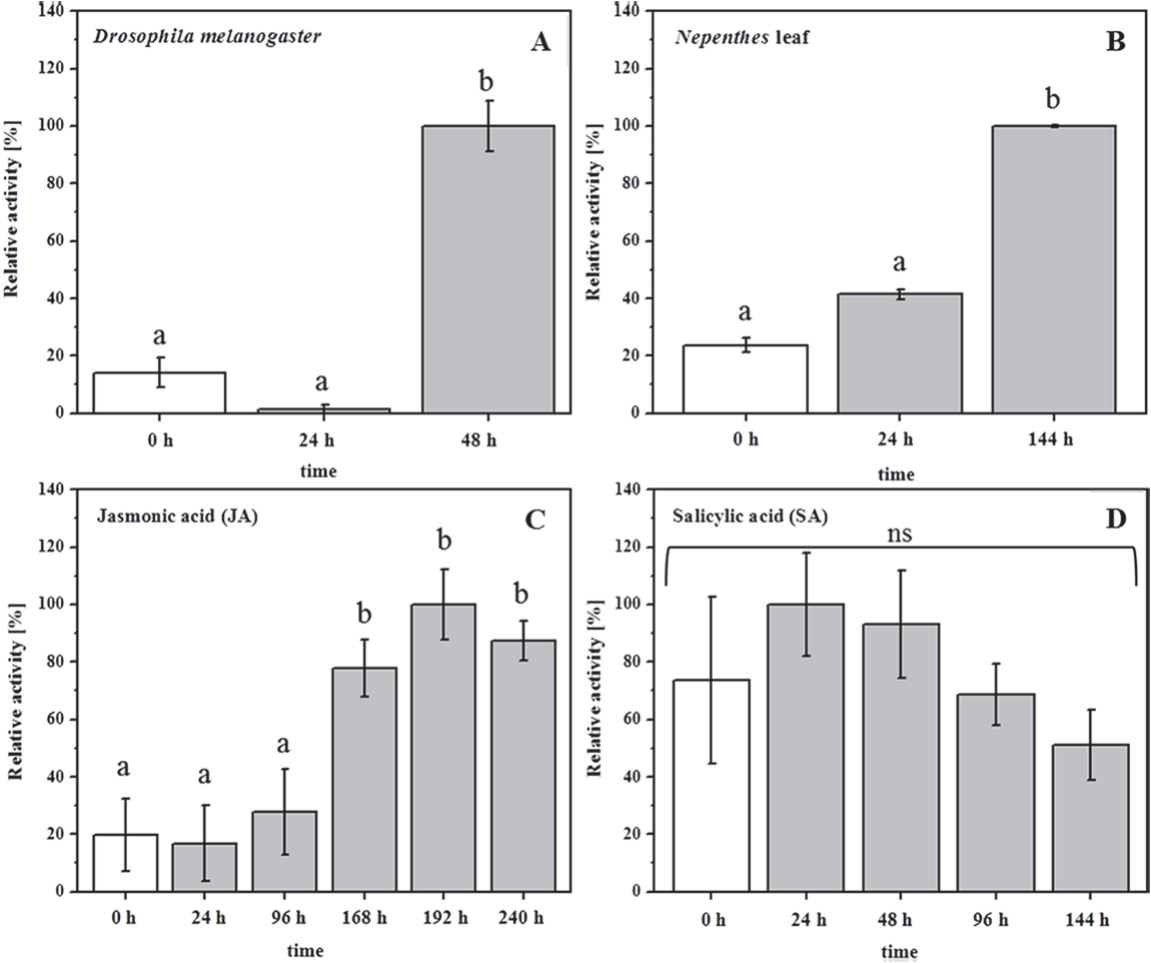
Induction of nepenthesin protease activity in *Nepenthes mirabilis* pitcher fluid. Proteolytic activity was measured with PFU-093 substrate at different time points after various treatments: **A** supplementation with *D*. *melanogaster* (*n* = 3); **B** supplementation with *Nepenthes* leaf (*n* = 2); **C** injection of jasmonic acid (200 μM end concentration) (*n* = 3) **D** injection of salicylic acid (end concentration 200μM) (*n* = 4). Statistics employed one-way ANOVA, P<0.05, Post Hoc Tests_*SNK* **(A, C, D),** Dunnett T3 **(B).**

Up to now, the induction of hydrolytic enzymes in the pitcher was investigated only indirectly by gene induction analyses of the pitcher tissue [5,6,13,33]. Now, we are able to follow such dynamic processes simply by determining the Nep protease activity. Such analyses require pitchers of *N*. *mirabilis* and *N*. *alata* to first be treated by supplementing them with prey such as *D*. *melanogaster*. This treatment resulted in a significant increase in proteolytic activity after 48 h (Fig. 7A). With some delay, the same effect was visible after supplementing the pitchers with *Nepenthes* leaf material (Fig. 7B). Although the latter finding might suggest a kind of ‘cannibalism’ in *Nepenthes*, this was not surprising because it is known that carnivorous plants actually take whatever they get for their nutrition. For example, *N*. *ampullaria* is specialized to capture leaf litter from the canopy above [34]. With any type of organism that falls in the trap all sources of nutrients become available for the plant and justify the induction of various hydrolyzing enzymes.

Recently, it has been shown for different carnivorous plant genera that defense-related phytohormones are involved in the trapping process, e.g. for *Drosera capensis* [35,36] as well as in the digestion process, e.g. in *D*. *muscipula* [37,38]. In order to follow up those studies, we investigated the effects of phytohormones in *Nepenthes* on protease activity. Interestingly, when phytohormones were added to the pitcher, only the addition of jasmonic acid (JA), not salicylic acid (SA), significantly increased the Nep- activities (Fig. 7C and 7D). These results support the hypothesis that carnivory in plants might have evolved from defensive reactions against pathogens or herbivores [7,36].

## Conclusion

Carnivorous plants of *Nepenthes*, unlike other plant carnivores, offer the possibility of working with sterile digestive fluids as long as the pitchers are closed or the open pitchers kept somewhat sterile by means of gauze. Here, a novel FRET-based method regarding *Nepenthes* plants was established, optimized and successfully applied by using the fluorogenic PFU-093 substrate for easy and rapid detection of protease activities in digestive fluids of *Nepenthes* species. The specificity of the substrate for aspartic proteases provides a means of unravelling the processes involved in prey digestion in carnivorous pitcher plants and possibly in other carnivorous plants. The ability to measure induced protease activity in pitcher fluids is much more reflecting the digestive process as quantifying transcripts of the corresponding genes.

## Supporting Information

S1 File Fig. A in S1 File. FRET-peptide,-method and putative cleavage sites. **a** Simplified structure of the artificial substrate PFU-093, according to Kaman et al. [19]. The substrate contains a fluorescein isothiocyanate (FITC) as fluorophore (F) and Lysin-Dabcyl (LysDbc) as its quencher (Q) connected by a two valine (Val) bridge. The close vicinity of Q to F quenches the fluorescence. **b** Putative proteolytic cleavage sites of PFU-093 substrate either between the two Val or between the last Val and the LysDbc, resulting in cleavage products: 1) FITC-Val, 2) Val-LysDbc, 3) LysDbc; **c** predicted molecular masses of these products: 1) 619.68 g/mol, 2) 496.28 g/mol and 3) 397.47 g/mol. **Fig. B in S1 File**. **Substrate/product fluorescence dependence on different pH values. a** Pitcher fluid was incubated with PFU-093 substrate and pure water at 42°C for 10 h. 50 μl each of this mixture were given in different wells and mixed with additional 50 μl of 30 mM buffer solutions to adjust the final pH (2, 4, 6, 8, 10, black dots), before fluorescence was measured. Control with water instead of buffer was included, representing the original fluorescence. Arrows indicate the pH-depending change of fluorescence. **b** Digestive fluid and PFU-093 substrate were incubated in 1 mM citrate buffer pH 4, for 10 h at 42°C. Subsequently, the mixture was split up and pH was adjusted by topping with either 30 mM phosphate buffer, pH 8, (dark grey bar) or 30 mM citrate buffer, pH 4, (striped) before fluorescence measurement. **Fig. C in S1 File. *N*. *mirabilis* nepenthesin I, II (*Nm*NepI/ II) protein alignment compared to *N*. *gracilis* (*Ng*) and *N*. *alata* (*Na*) nepenthesin amino acid sequences**. The four levels of shading used are: *blue* > 80% sequence identity, *mid-blue* > 60% identity, *light blue* > 40% identity and *no shading* < 40%. Regions of predicted signal peptides and propeptides are named and the endings marked by a black stroke. Aspartic acid residues of the active center are indicated by a small black box and the flap tyrosine residue by a small green box, both above the sequences. The cysteine residues are represented through the colors: yellow, orange, green, light green, red and light red. The colored pairing of the residues show the disulphide bond arrangements in the primary structures of nepenthesin. **Fig. D in S1 File**. **Western blot of recombinant *N*. *mirabilis* nepenthesin II.** *Nm*NepII/Sf9 was expressed in Sf9 insect cell line, using an anti-V5 horseradish peroxidase antibody and ECL for detection Lanes represent 1) lysate, negative control, 2) culture medium, negative control, 3) lysate, positive control, 4) culture medium, positive control (CAT, catalase of 34 kDa); the blot also contains duplicates (clone 1 and 2) shown by the lanes 5) *Nm*NepII/Sf9, clone 1, lysate, 6) *Nm*NepII/Sf9, clone 1, culture medium, 7) *Nm*NepII/Sf9, clone 2, lysate, 8) *Nm*NepII/Sf9, clone 2, culture medium, all in comparison to the Sf9 cell line stably expressing *Nm*NepII/Sf9, pointed out with 5 μl (lane 9) and 10 μl (lane 10), respectively. **Table A in S1 File**. **List of primer sequences used in this study**.
(PDF)
