# Supplementary material for: Nepenthesin Protease Activity Indicates Digestive Fluid Dynamics in Carnivorous Nepenthes Plants
Source: PLoS One. 2015 Mar 9;10(3):e0118853. doi: 10.1371/journal.pone.0118853 (PMC4353617; doi:10.1371/journal.pone.0118853)
Supplement: S1 File — FRET-peptide,-method and putative cleavage sites. a Simplified structure of the artificial substrate PFU-093, according to Kaman et al. [19]. The substrate contains a fluorescein isothiocyanate (FITC) as fluorophore (F) and Lysin-Dabcyl (LysDbc) as its quencher (Q) connected by a two valine (Val) bridge. The close vicinity of Q to F quenches the fluorescence. b Putative proteolytic cleavage sites of PFU-093 substrate either between the two Val or between the last Val and the LysDbc, resulting in cleavage products: 1) FITC-Val, 2) Val-LysDbc, 3) LysDbc; c predicted molecular masses of these products: 1) 619.68 g/mol, 2) 496.28 g/mol and 3) 397.47 g/mol. Fig. B in S1 File. Substrate/product fluorescence dependence on different pH values. a Pitcher fluid was incubated with PFU-093 substrate and pure water at 42°C for 10 h. 50 μl each of this mixture were given in different wells and mixed with additional 50 μl of 30 mM buffer solutions to adjust the final pH (2, 4, 6, 8, 10, black dots), before fluorescence was measured. Control with water instead of buffer was included, representing the original fluorescence. Arrows indicate the pH-depending change of fluorescence. b Digestive fluid and PFU-093 substrate were incubated in 1 mM citrate buffer pH 4, for 10 h at 42°C. Subsequently, the mixture was split up and pH was adjusted by topping with either 30 mM phosphate buffer, pH 8, (dark grey bar) or 30 mM citrate buffer, pH 4, (striped) before fluorescence measurement. Fig. C in S1 File. N. mirabilis nepenthesin I, II (NmNepI/ II) protein alignment compared to N. gracilis (Ng) and N. alata (Na) nepenthesin amino acid sequences. The four levels of shading used are: blue > 80% sequence identity, mid-blue > 60% identity, light blue > 40% identity and no shading < 40%. Regions of predicted signal peptides and propeptides are named and the endings marked by a black stroke. Aspartic acid residues of the active center are indicated by a small black box and the flap tyrosine residu [file pone.0118853.s001.pdf]

<u>Supporting Information</u>

Nepenthesin protease activity displays the digestive fluid dynamics in carnivorous *Nepenthes* plants

Franziska Buch, Wendy E. Kaman, Floris J. Bikker, Ayufu Yilamujiang and Axel Mithöfer

Figures A-D

Table A

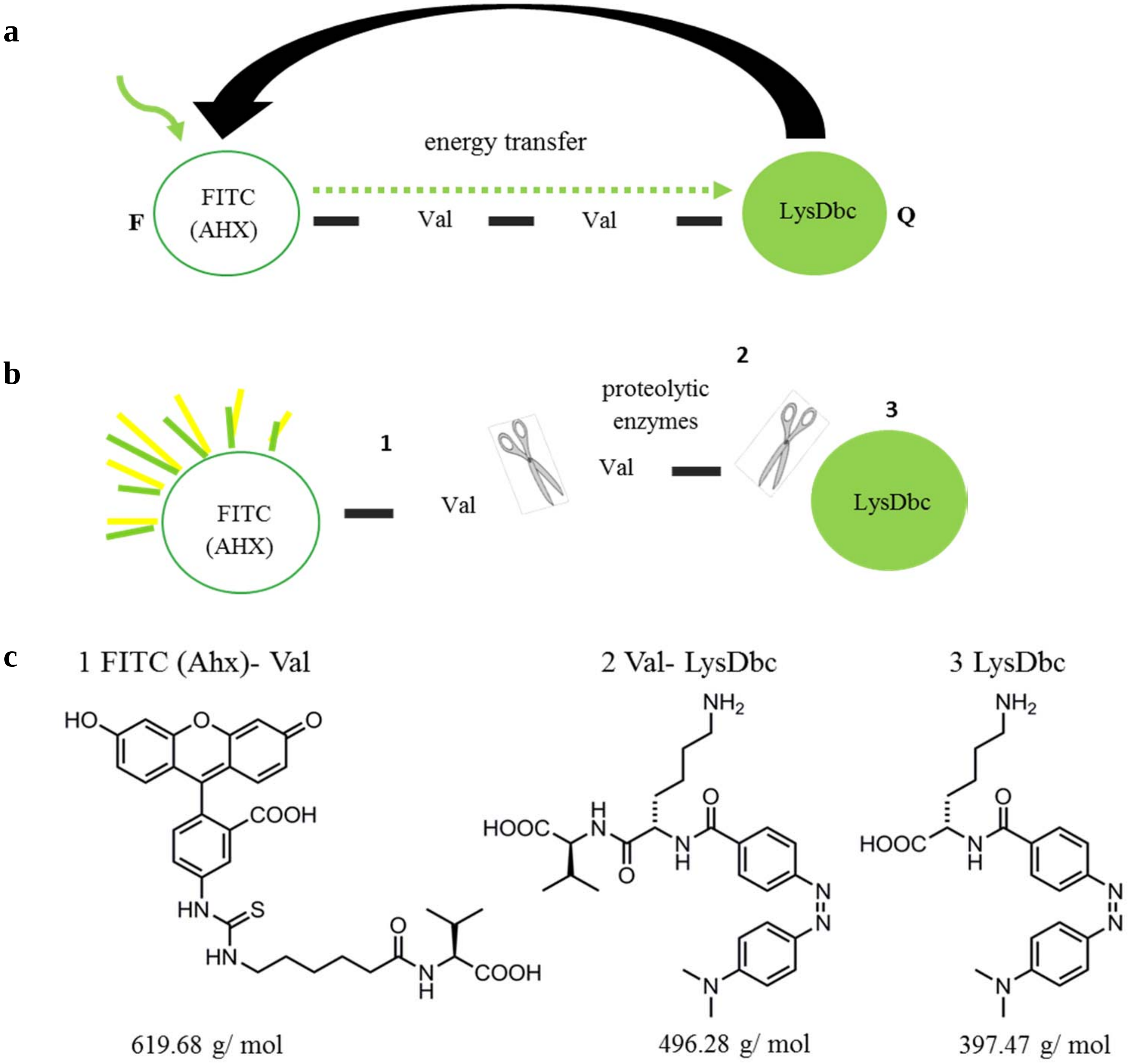

Figure A. **FRET-peptide, -method and putative cleavage sites.**

a

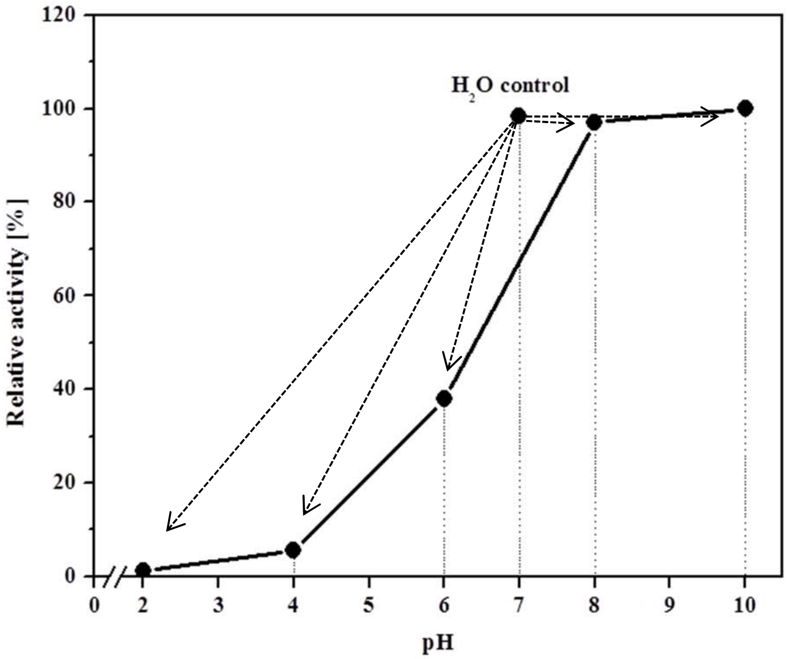

b

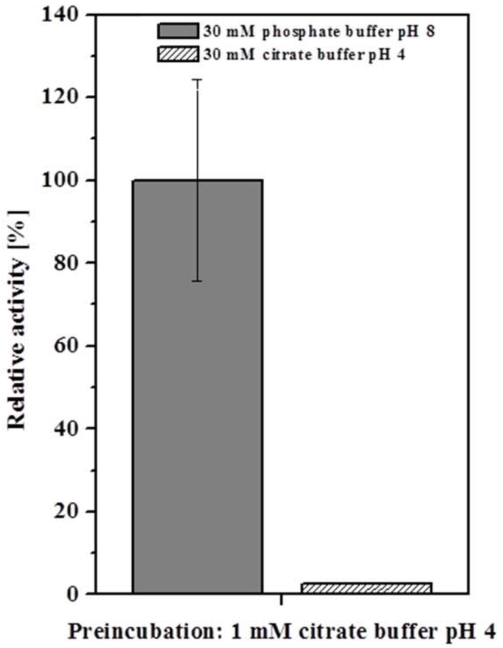

Figure B. **Substrate/ Product fluorescence dependence on different pH values.**

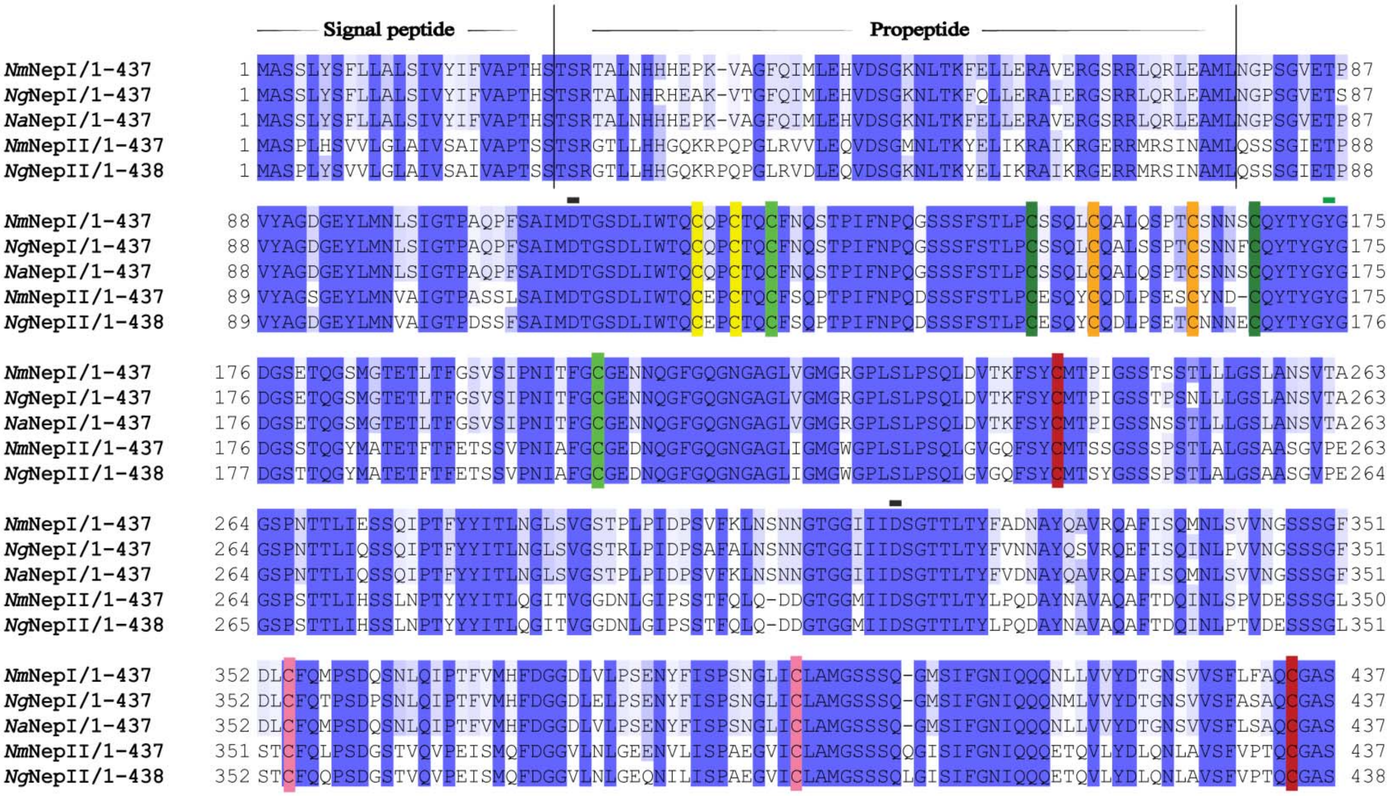

Figure C. ***N. mirabilis*** **nepenthesin I, II (*Nm*NepI/ II) protein alignment compared to *N. gracilis* (*Ng*) and *N. alata* (*Na*) nepenthesin amino acid sequences**.

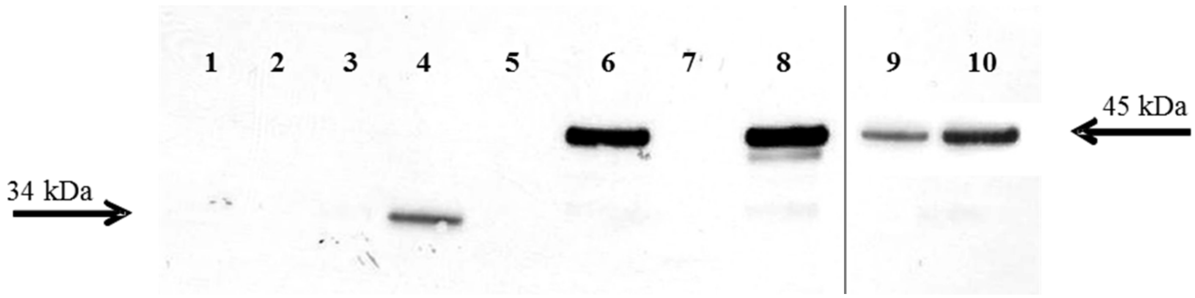

Figure D. **Western blot of recombinant *N. mirabilis* nepenthesin II.**

Table A. List of primer sequences used in this study.

1_NepI_forward_partialSequence	5‘ CGGGCAAAAACTTAACCAAA 3‘
2_NepI_reverse_partialSequence	5‘ ACCGTCAAAATGCATCACAA 3‘
3_NepII_forward_partialSequence	5‘ TTCCTTGCGAGAGCCAGTAT 3‘
4_NepII_reverse_partialSequence	5‘ GCTGCTGGATATTCCCAAAA 3‘
5_NepI_3‘-RACE	5‘ GTGCTGTCGAAAGGGGGAGTCGTAG 3‘
6_NepI_5‘-RACE	5‘ CGGAGAAAGGTTGTGCCGGAGTC 3‘
7_NepII_3‘-RACE	5‘ CTCCTCACCCAGCACTCTCGCACTT 3‘
8_NepII_5‘-RACE	5‘ ACCGCATTGTAAGCGTCTTGTGGAAG 3‘
9_NepI_forward_pMIB/V5-HisAvector	5’ TAATGCATGCTAACGTCAAGAACAGCTCTCAATC 3’
10_NepI_reverse_pMIB/V5-HisAvector	5’ ATTATCTAGACGACGCACCACATTGAGCAC 3’
11_NepII_forward_pMIB/V5-HisAvector	5’ TAATGCATGCTAACCTCAAGAGGAACCCTTCTTCATCAT 3’
12_NepII_reverse_pMIB/V5-HisAvector	5’ ATTATCTAGACGACGCACCACACTGAGTAGGAACGAA 3’
